# Metabolic reprogramming associated with progression of renal ischemia reperfusion injury assessed with hyperpolarized [1-^13^C]pyruvate

**DOI:** 10.1038/s41598-020-65816-1

**Published:** 2020-06-02

**Authors:** Per Mose Nielsen, Haiyun Qi, Lotte Bonde Bertelsen, Christoffer Laustsen

**Affiliations:** MR Research Centre, Department of Clinical Medicine, Aarhus University, Aarhus, Denmark

**Keywords:** Kidney, Metabolism, Acute kidney injury

## Abstract

Acute kidney injury is a major clinical challenge affecting as many as 1 percent of all hospitalized patients. Currently it is not possible to accurately stratify and predict the outcome of the individual patient. Increasing evidence supports metabolic reprogramming as a potential target for new biomarkers. Hyperpolarized [1-^13^C]pyruvate imaging is a promising new tool for evaluating the metabolic status directly in the kidneys. We here investigate the prognostic potential of hyperpolarized [1-^13^C]pyruvate in the setting of acute kidney injury in a rodent model of ischemia reperfusion. A significant correlation was found between the intra-renal metabolic profile 24 hours after reperfusion and 7 days after injury induction, as well as a correlation with the conventional plasma creatinine biomarker of renal function and markers of renal injury. This leads to a possible outcome prediction of renal function and injury development from a metabolic profile measured *in vivo*. The results support human translation of this new technology to renal patients as all experiements have been performed using clinical MRI equipment.

## Introduction

Acute kidney injury is one of the leading causes of end-stage kidney disease, a disease with a mortality rate estimated to 2 million per year worldwide^1–3^. The kidney is a well perfused organ, and it consumes as much as 20% of the bodies total available oxygen. The high oxygen consumption occurs to maintain the high energetic burden of transporting water and waste products against the solute concentration gradients. This in combination with the heterogenetic tissue composition of the kidney renders the kidney especially vulnerable to hypoxic injury^4,5^. This has led to the unifying mechanism, that hypoxia is a key contributor in the development of renal disease^6,7^. A recent study demonstrated that 60 minutes (min) of ischemia in the kidneys, lead to a fully hypoxic condition, while 30 min of ischemia in the kidneys did not show any sign of reduction in the oxidative metabolism *in vivo*, albeit a reduced *ex vivo* pyruvate dehydrogenase (PDH) activity was observed^5^.

A recent report has identified a mismatch in the redox balance (NADH/NAD + ) in concern with sepsis induced acute kidney injury (AKI), even in the presence of sufficient oxygen in a rat model^8^. However, both the AKI induction method, reperfusion time and animal model lead to high heterogeneity in both the injury outcome and redox balance consequences. We have previously shown an elevation in the redox capacity together with a reduction in glycolytic capacity^5^ when working in this AKI model. This is supported by Zager *et al*. and Claworthy *et al*.^9,10^, with studies performed in the early stages as well as in the ischemia reperfusion injury (IRI) induced AKI (1–48 hours of reperfusion). On the contrary Baligand *et al*.^11^ also reported a reduction in glycolytic capacity but with a reduced redox capacity instead. This illustrates that comparisons between AKI models must be performed with caution. A new imaging method, the so-called hyperpolarized carbon-13 (^13^C) magnetic resonance imaging (MRI) method, has proven particularly well-suited for renal investigations^12^, as researchers and clinicians can use it to follow the metabolic alterations minimally-invasively in real-time only seconds after injection of ^13^C-labelled metabolites^13–18^. The method has been used to investigate the pseudo-hypoxic condition in diabetic nephropathy in which anaerobic metabolism is elevated despite sufficient oxygen supply^7,19^ and to utilize this metabolic derangement as a novel therapeutic target for treatment of diabetic kidney disease^20–22^. Thus, hyperpolarized ^13^C MRI is ideally suited to investigate the pseudo-hypoxic phenomena associated with diabetes together with the metabolic alterations seen in ischemia reperfusion injury.

The hyperpolarized [1–^13^C]pyruvate allows for quantification of the pyruvate flux to its products [1–^13^C]lactate, [1–^13^C]alanine and ^13^C-bicarbonate. The conversion of pyruvate-to-lactate is catalyzed by the enzyme lactate dehydrogenase (LDH), a reaction that is considered to be indicative of the glycolytic activity (anaerobic metabolism). The conversion of pyruvate-to-alanine is catalyzed by the enzyme alanine transferase (ALT), a reaction that is considered to be indicative of the amino acid requiements. The production of bicarbonate (HCO_3_-) from pyruvate is catalyzed by the mitochondrial enzyme pyruvate dehydrogenase (PDH) and the enzyme carbonic anhydrase (CA), a reaction that is considered to be indicative of the oxidative metabolism (oxidative phosphorylation)^12,23^. The balance between the anaerobic and the aerobic pathways, can thus be represented by the ratio between the two metabolic products lactate and HCO_3_- respectively. This measure has previously been shown to evaluate ishcmic injury in both heart and kidneys^5,10,11,24,25^, and thus represents an opportunity to evaluate the time course of the change that occur during AKI.

Clatworthy *et al*. reported no change in lactate-to-bicarbonate ratio in an Folic acid induced AKI model^10^, whereas Baligand *et al*. found a reduction in the bicarbonate-to-lactate ratio and elevation of the lactate-to-pyruvate ratio in a ischemia reperfusion model^11^, these contradictions might be caused by differences in AKI induction models or their timing. In a previous study in a similar I/R model we found a reduction in lactate-to-pyruvate ratio, but with an elevation of the lactate-to-bicarbonate ratio indicating reduced overall metabolic capability but a preference towards anaerobic metabolism^5^, which is in agreement with Baligand *et al*.^11^.

In this study we question if the acute and prolonged metabolic consequences (lactate-to-bicarbonate) associated with mild ischemia reperfusion injury (20 min, 30 min and 40 min), are detectable in the early stages following reperfusion (30 min ischemia imaged up to 60 min), and secondly if this derangement is dependent on the extend of the injury (30 ± 10 min ischemia imaged at 1 and 7 days) and as such can be used to predict the outcome of the long-term metabolic patterns associated with AKI.

## Materials and Methods

### Rat model

**Figure 1:**
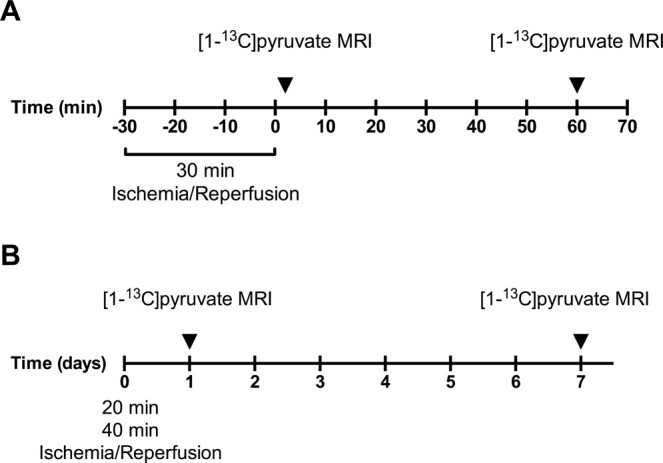
Illustration of the experimental design. (**A**) Ischemia was induced in the scanner for 30 min. 1 minute after clamp release [1–^13^C]pyruvate MRI was performed. 60 min after clamp release another [1–^13^C]pyruvate MRI scan was performed. (**B**) Two groups of 20 min and 40 min of ischemia were produced. The animals were scanned after 1 day and again after 1 week.

Two surgical procedures were utilized to investigate firstly the immediate effect following reestablisment of the blood flow to the kidney (Fig. 1A) and secondly the long term effects of the ischmia (Fig. 1B). In the first procedure an incision was performed in male Wistar rats (n = 6) weighing between 200–233 g and the left renal artery was carefully dissected. A balloon artery cuff was placed around the left renal artery. After surgery the rodents were placed in the MR scanner, and a rectal temperature of 37 °C was maintained. Respiration and oxygen saturation were monitored in the scanner. The balloon cuff was inflated and ischemia was induced and maintained for 30 min. After 30 min the cuff was deflated and a [1–^13^C]pyruvate MRI scan protocol was performed after 1 and 60 min of reperfusion (Fig. 1A). In the second surgical procedure male Wistar rats (n = 6 in each group) weighing between 210–245 g were placed on a heating pad to maintain a rectal temperature of 37 °C. We performed a surgical incision in the abdomen, and the left renal artery was carefully dissected. We placed a non-traumatic clamp on the left artery for 20 min and 40 min respectively in each group to induce ischemia. After release of the clamp, reperfusion was visually confirmed. The incision was sutured separately in muscle tissue and skin^5,26,27^. The animals were scanned after 1 day and 7 days (Fig. 1B). During surgery and scan sessions, the animals were anesthetized with Sevoflurane (induction 8%, sustained 6%) mixed with air (2 l/min). At the beginning of surgery, Temgesics was given subcutaneously as analgesics (0.05 mg/kg) and was then supplied in the drinking water (0.3 mg/ml) for 48 hours. To maintain post-operative water balance, 2 ml of isotonic salt water was injected subcutaneously in the beginning of the operation^5,26,27^. The study was carried out in accordance with Danish National Guidelines for animal care, and was approved by the Danish Animal Experiments Inspectorate under the Danish Veterinary and Food Administration (License no. 2014–15–0201–00327).

### Hyperpolarization experiments

Tail vein catheterization was performed for administration of hyperpolarized [1–^13^C]pyruvate. Temperature, peripheral capillary oxygen saturation and respiration rate were monitored throughout the experiment. [1–^13^C]pyruvate was polarized in the Spinlab (GE Healthcare, Brøndby, Denmark). Polarization was performed as previously described^5^. Each animal was injected with 1.5 ml (125 mM) hyperpolarized [1–^13^C]pyruvate. MR scans were performed in a 3 T clinical MR system (GE Healthcare, Brøndby, Denmark) equipped with a dual tuned ^13^C/^1^H volume rat coil (GE Healthcare, Brøndby, Denmark). A slice-selective ^13^C IDEAL spiral sequence was used for hyperpolarized ^13^C-pyruvate imaging acquiring images every 5 second (sec). The sequence was initiated 20 sec. after the start of injection with the following parameters: flip angle = 10°, 11 IDEAL echoes and one initial spectrum per IDEAL encoding, TR/TE/ΔTE = 100 ms/0.9 ms/0.9 ms, FOV = 80 × 80 mm^2^, 5 × 5 mm real resolution and an axial slice thickness of 15 mm covering both kidneys. Acquired ^1^H and ^13^C images were converted to dicom files. Regions of interest (ROI’s) were placed around each kidney in Osirix. Hyperpolarized MR metabolic kinetic analysis was performed by ratiometric analysis according to previous reports^28^.

### Tissue and blood sampling

Prior to the MRI scan sessions blood samples were drawn from the tail vein catheter and placed in heparinized tubes. Blood samples were centrifuged and the plasma samples were stored at −80 °C. After the last MRI scan protocol, the animals were sacrificed and tissue samples of the right and left cortex and inner medulla (IM) were collected and snap frozen in liquid nitrogen. Tissue samples were stored at −80 °C. Plasma creatinine was measured using an enzymatic approach using a Roche Cobas 6000 analyzer, following the vendors instructions (Roche Diagnostics, Hvidovre, Denmark).

### RNA extraction and qPCR

IM was homogenized, and total RNA was isolated using a Nucleospin RNA II kit (Stratagene, AH diagnostics, Aarhus, DK) following manufacturer’s instructions^5,26,27^. RNA purity and concentration was measured by spectrophotometry. cDNA synthesis was performed on 0.5 µg RNA with the Revertaid First Strand cDNA synthesis kit (Thermo Fisher Scientific, Hvidovre, DK), following manufacturer’s instructions^5,27^. Preparation of samples for qPCR was performed using Maxima SYBR Green qPCR master mix (ThermoFisher Scientific, Hvidovre, DK), following manufacturer’s instructions^5,26,27^. The qPCR comprised 40 cycles of denaturation for 30 sec. at 95 °C followed by annealing and synthesis for 1 min at 60 °C. Primer sequences are described in Table 1.

**Table 1:** qPCR primers.

Gene:	Forward primer sequence:	Reverse primer sequence:
**18 s**	5′-CAT GGC CGT TCT TAG TTG-3′	5′-CAT GCC AGA GTC TCG TTC-3′
**LDH**	5′-AAT AAT ACG TGA AAT GTA AGA-3′	5′-TTT TCC CTT GGC ATG CAC TTG-3′
**PDH**	5′-TCC ACT CCT TGTAGC TGC AAC-3	5′-GAG AAC CCA CCA CCC CATG-3′
**KIM-1**	5′-CCA CAA GGC CCA CAA CTA TT-3′	5′-TGT CAC AGT GCC ATT CCA GT-3′
**NGAL**	5′-GAT CAG AAC ATT CGT TCC AA-3′	5′-TTG CAC ATC GTA GCT CTG TA-3′
**MCT1**	5′-CTC TGG GCG CCG CGA GAT AC-3′	5′- CAA CTA CCA CCG CCC AGC CC-3′
**MCT4**	5′- CCA GGC CCA CGG CAG GTT TC-3′	5′- GCC ACC GTA GTC ACT GGC CG-3′

Primers used for qPCR.

### Biochemical assays

LDH and PDH activity and lactate and fumarase activity assays were performed according to the manufacturer’s instructions (Sigma Aldrich, Brøndby, Denmark) with few alterations. Tissue was homogenized in assay buffer specific for each activity kit, the solution was centrifuged and the supernatant was used for assay quantification. Analysis was performed on 386 well plates. Initially a full spectrum absorbance measurement was performed to find the optimal absorbance peak (PDH 410 nm, LDH 458 nm, lactate 579 nm, fumarase 458 nm). Hereafter the absorbance protocol followed manufacturer’s recommendation. Activity measurements on tissue were normalized to protein content. Fumarase activity was measured directly on plasma and urine fractions according to manufacturer’s instructions (Merck, Brøndby, Denmark).

### Statistics

All data are presented as means ± SEM. All statistical analysis was performed in GraphPad Prism 6. Data were analyzed by either a one-way-ANOVA with repeated measures or a two-way-ANOVA repeated measures. A value of P < 0.05 (*) was considered statistically significant.

## Results

### Acute compensatory metabolic regulation maintains aerobic and anaerobic balance

**Figure 2:**
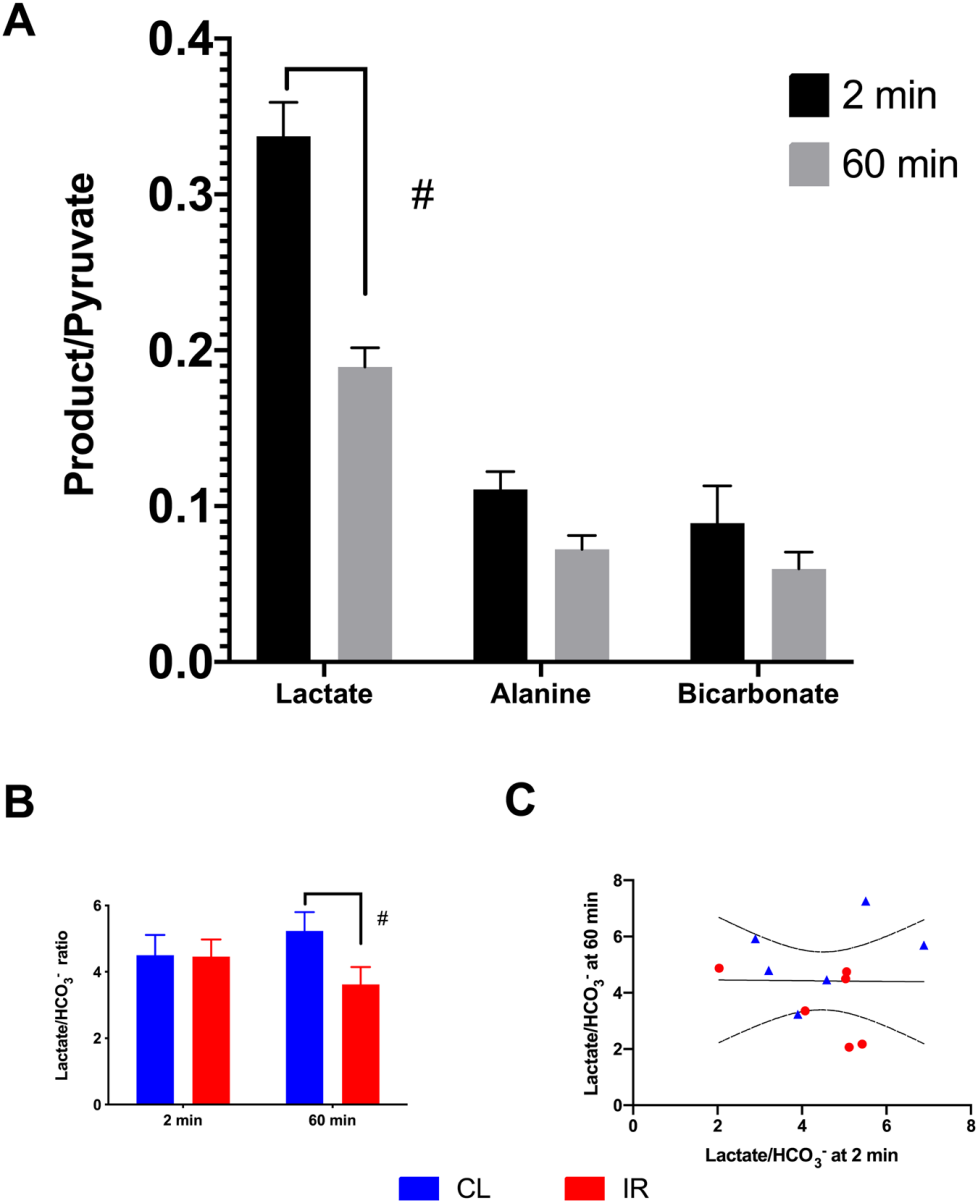
Acute metabolic effects 2 min and 60 min after reperfusion in the ischemic kidney. (**A**) A significant different metabolic profile (*lactate-to-pyruvate, alanine-to-pyruvate and bicarbonate-to-pyruvate*) between 2 min and 60 min was found p = 0.031*, as well as a significant interaction term (Time x Metabolites, p = 0.0004^#^). Multiple-comparison (*Lactate-to-pyruvate****)*** p = 0.016* (*Alanine-to-pyruvate*) p = 0.16; (*Bicarbonate-to-pyruvate*) p = 0.77. (**B**) The balance between the anaerobic and the aerobic metabolism between the contralateral and ischemic kidney (*Lactate-to-Bicarbonate*) p = 0.14 for kidney, p = 0.95 for time and for interaction (Time x Kidney, p = 0.02^#^) as seen with a counter difference in contralateral and ischemic kidney at 60 min p = 0.005. (**C**) No correlation between the lactate-to-bicarbonate ratio was seen between 2 min and 60 min (0.0001 = R^2^, p = 0.97). Data is represented as mean ± SEM and statistical significance p < 0.05 is represented as * and ^#^.

Acute alterations in the ischemic re-perfused kidneys overall metabolic phenotype were seen between the ischemic/early perfusion stage (2 min) and after 1 hour of perfusion, showing a compensatory mechanism in the contralateral kidney 60 min after reperfusion (Fig. 2A-B). The acute change in the lactate-to-bicarbonate ratio 60 min after reperfusion was not correlated with the early signature 2 min after reperfusion (Fig. 2C). The acute metabolic reprogramming seen at 60 min was driven by post release compensation in the contralateral kidney as well as downregulation of the lactate-to-bicarbonate balance in the ischemic kidney.

### Ischemic insult mediated anaerobic adaptation over time in both the contralateral and the ischemic kidney

**Figure 3:**
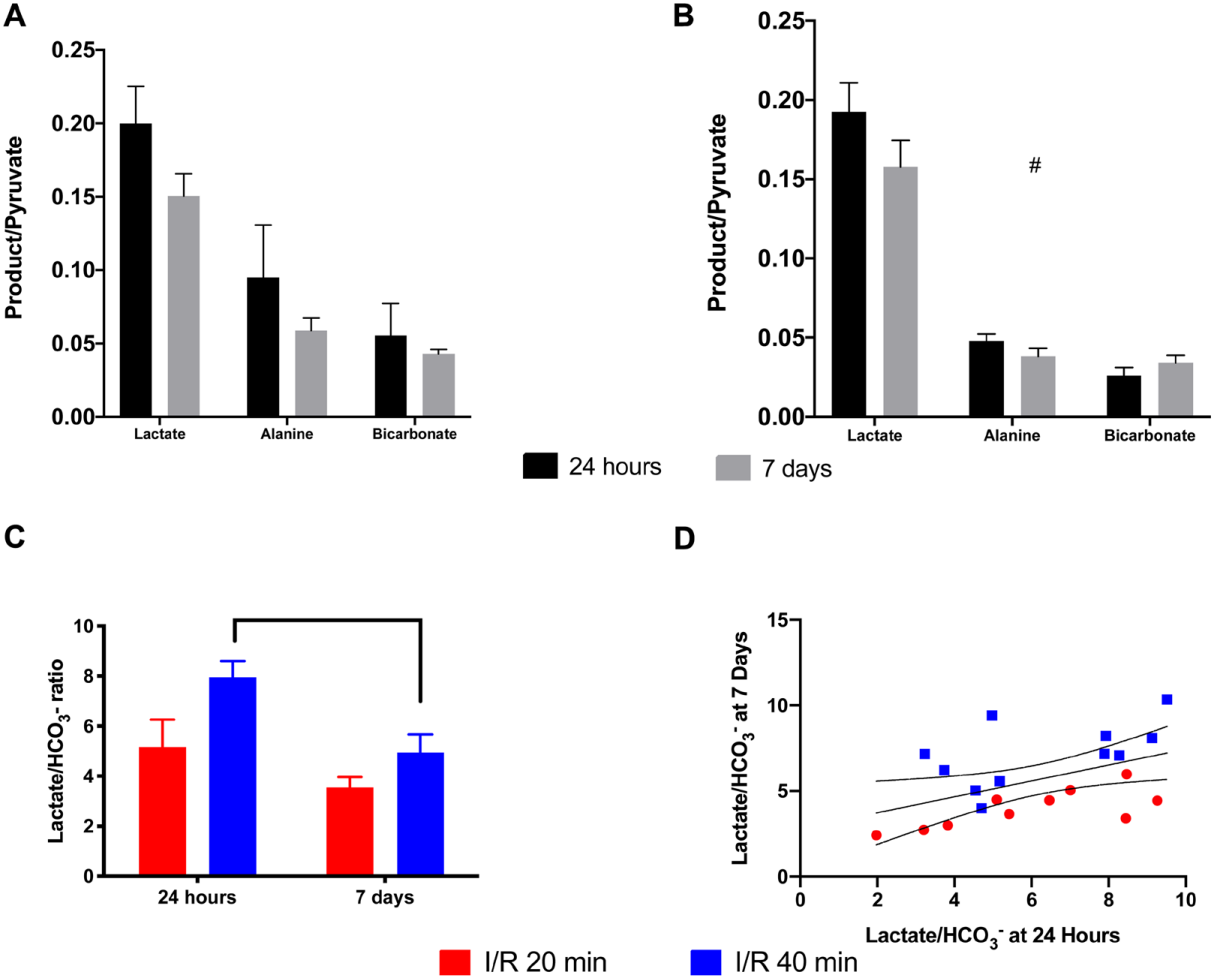
The metabolic effects of 20 min and 40 min ischemia time 24 hours and 7 days after reperfusion in the ischemic kidney. (**A**) 20 min ischemia did not result in a significant different metabolic profile (*lactate-to-pyruvate, alanine-to-pyruvate and bicarbonate-to-pyruvate*) between 24 hours and 7 days p = 0.38, nor interaction term (Time x Metabolites, p = 0.16). (**B**) A numerically lower metabolic conversion was seen with 40 min ischemia (*lactate-to-pyruvate, alanine-to-pyruvate and bicarbonate-to-pyruvate*) between 24 hours and 7 days, although it did not reach statistical significance p = 0.06, while a significant interaction term (Time × Metabolites, p = 0.03^#^) was found. (**C**) This tendency towards a reduction over time (*Lactate-to-Bicarbonate*) was similar in both groups Time p = 0.01 and Ischemia time (20 versus 40 min) p = 0.03. (**D**) A positive correlation was found between the 24 hours (*Lactate-to-Bicarbonate*) and the 7 days (*Lactate-to-Bicarbonate*) ratio (R^2^ = 0.24, p = 0.02*).

The *in vivo* response that was seen 24 hours and 7 days following ischemic injury showed a similar tendency towards a general reduction of the overall metabolism in the ischemic kidney as well as a compensatory increased anaerobic metabolism shown by increased lactate production when compared to the aerobic metabolism, shown by CO_2_ and HCO_3_- production. Both 20 min and 40 min ischemia in the kidney results in a tendency towards a metabolic reprogramming from 24 hours to 7 days, with a statistically significant shift observed in the 40 min group (Fig. 3A-C). The metabolic phenotype at 24 hours, with reduced lactate-to-bicarbonate ratio, is positively correlated with the lactate-to-bicarbonate ratio at 7 days and well separated by severity (Fig. 3D).

### Early metabolic programming correlates with ischemic severity following reperfusion and thus represents a novel theranostic target

**Figure 4:**
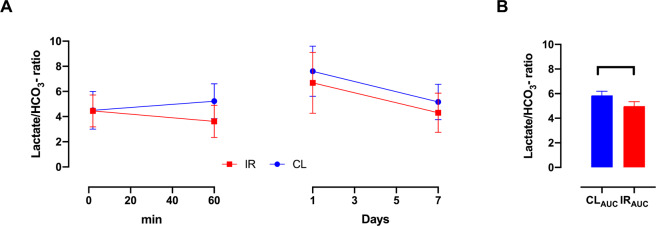
**(A)** (*Lactate-to-Bicarbonate*) Looking at the data from 2 min – 7 days, seems to indicate a rapid and consistent downregulation. (**B**) A significant reduced (*Lactate-to-Bicarbonate*) area under curve (AUC) of the ischemic kidney was found p = 0.005*.

**Figure 5:**
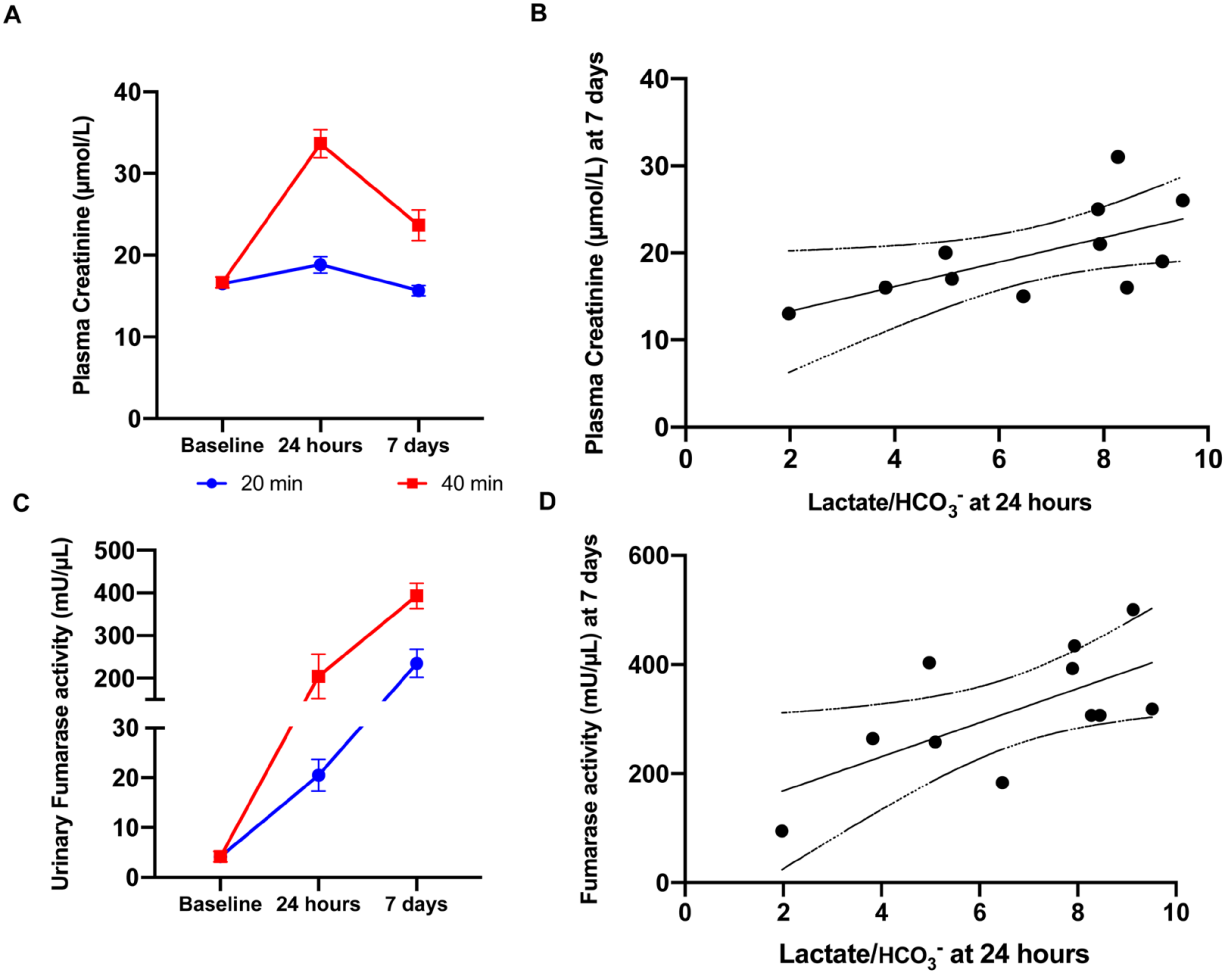
Plasma creatinine and urinary fumarase activity and correlations with lactate-to-bicarbonate in 20 min and 40 min ischemia time 24 hours and 7 days after reperfusion as well as 2 min and 1 hour after ischemia. (**A**) Plasma Creatinine levels were significantly elevated between 20 and 40 min of ischemia p = 0.0003. Reperfusion time p < 0.0001 as well as the interaction term p < 0.0001 were significant. (**B**) The correlation between endpoint plasma creatinine (7 days of reperfusion) and *Lactate-to-Bicarbonate* were significant p = 0.04, r^2^ = 0.39. (**C**) Urinary fumarase levels were significantly elevated between 20 and 40 min of ischemia p < 0.0001. Reperfusion time p = 0.0018 as well as the interaction term p = 0.0029 were significant. (**D**) The correlation between endpoint urinary fumarase activity (7 days of reperfusion) and *Lactate-to-Bicarbonate* were significant p = 0.03, r^2^ = 0.43.

A positive correlation was found in the lactate-to-bicarbonate ratio between 24 hours and 7 days. While no such correlation was found between the perfusion stage (2 min) and 60 min. It is however unclear if there exists an earlier time point for predicting the progression of the ischemic injury. By looking at the 20 min and 40 min group, one group with a large variance covering the 30 min acute insult, we compared the overall metabolic pattern from the initial 2 min – 7 days between the ischemic and contralateral kidney (Fig. 4A-B). This combination shows a significant change already at 60 min which persists throughout the 7 days. These findings are supported by a tendency towards a correlation between the lactate-to-bicarbonate ratio and the LDH activity and MCT-4 expression (Table 2), as well as the cell death/damage and kidney functional biofluid markers fumarase activity and plasma creatinine respectively (Table 2, Fig. 5A-D).

**Table 2:** *In vivo* anarobic-to-aerobic balance correlations with endpoint biomarkers.

24 hours *in vivo* versus end-point correlation	Lactate/HCO_3_^−^	
	*R*^2^	*p-values*
**Oxygen metabolism**		
LDH expression	0.047	0.53
LDH activity	0.279	0.09#
PDH expression	0.059	0.47
PDH activity	0.013	0.73
Lactate concentration	0.048	0.52
MCT-1	0.139	0.26
MCT-4	0.296	0.08#
**Damage Markers**		
NGAL	0.157	0.23
KIM-1	0.152	0.24
Plasma Fumarase activity	0.346	0.10#
Urine Fumarase activity	0.423	0.03*
**Functional markers**		
Plasma Creatine	0.390	0.04*

The anaerobic versus aerobic balance in metabolism(lactate-to-bicarbonate ratio) measured 24 hours after ischemia was compared to metabolic, injury and kidney function biomarkers. Urinary fumarase and plasma creatinine was significantly correlated with lactate-to-bicarbonate ratio, represented by *p < 0.05. LDH, MCT4 and plasma fumarase showed a non-significant tendency towards a correlation with the lactate-to-bicarbonate ratio, represented by ^#^p < 0.1.

## Discussion

The aim of this study was to investigate the acute and prolonged metabolic consequences associated with ischemia reperfusion injury, and to elucidate whether the early injury mediated metabolic reprogramming can predict the outcome of the injury. We found a metabolic reprogramming as early as 24 hours after injury predicting the outcome seven days later, as well as indications that this change can be seen as early as 1 hour after reperfusion.

A unilateral ischemia of 20–40 minutes is generally accepted to promote a mild to moderate ischemic injury, while 60 minutes or more of ischemia is needed for severe injury induction^29^. In a previous study we have shown that severe ischemic injury induction leads to a reduction in bicarbonate-to-pyruvate ratio as well as an elevation in lactate-to-pyruvate ratio^5^. This has led to the hypothesis that severe ischemic injury will lead to disruption and loss of mitochondria, which reduces the bicarbonate-to-pyruvate ratio, whilst moderate ischemic injury does not disrupt mitochondrial function. However, both are associated with decreased renal function as seen with increased levels of serum levels of creatinine, even with the compensation of the contralateral kidney. It is generally accepted that the mitochondria play a central role in injury induction in IRI^30^. This includes generation of reactive oxygen species especially in the reperfusion phase and as an integral factor leading to apoptosis and necrosis^31^. It is generally believed that the disruption of mitochondria inevitably will lead to cell death, whilst our former study showed an elevation of lactate-to-pyruvate ratio indicating that some cells are still present with anaerobic metabolic capabilities whilst losing the aerobic capabilities. You could argue that the elevated lactate-to-pyruvate ratio only represents circulating LDH activity or macrophage infiltration which is often found in concern with renal IRI^5,9^. However, in this study we find an elevation of lactate-to-pyruvate ratio whilst retaining the aerobic capabilities. This indicates a true anaerobic response to injury which is not only induced by the loss of LDH to the interstitium by cell wall disruption. We believe the anaerobic response leading to the elevation of lactate-to-pyruvate ratio is initiated by cells found in the distal parts of the kidney. The intra-renal oxygen gradient leads to a permanent hypoxic state in the distal areas which leads to cells specialized in anaerobic energy formation^32^.

All metabolites of ^13^C-pyruvate were found to be elevated in the ischemic/early reperfusion phase of 2 min although only the lactate-to-pyruvate ratio reached a significant level. These metabolites-to-pyruvate ratios might reflect a buildup of metabolites in the ischemic phase rather than a true metabolic phenotype at this early stage. This would also explain why no correlation between the lactate-to-bicarbonate ratios was found in the acute phase (between 2 min and 60 min of reperfusion). The correlations between lactate-to-bicarbonate ratios reflect a developing metabolic phenotype which follows injury induction and kidney function. Especially fumarase activity were found to correlate quite well with the phenotype. In a previous study we have shown how fumarase activity measured in urine and plasma fractions functions as a sensitive injury marker^26^. This verifies how the lactate-to-bicarbonate ratio phenotype reflects injury induction and developments as the ratio correlates with fumarase activity. We found a weak tendency towards an elevation of fumarase activity in the acute phase, while it was found to be highly upregulated 1 hour after reperfusion. This illustrates how most of injury induction happens in the reperfusion phase rather than the ischemic phase^4^. The fumarase activity is steadily increasing even 7 days after perfusion. This indicates that injury induction is still developing and that the repair phase has not reestablished complete kidney function yet which is supported by the elevated plasma creatinine levels. Further studies will be needed to fully understand this development. A limitation of this study is the lack of histological verification, which in combination with the used damage assays could have given even further information, future studies needs to address the mechanisms associated with metabolic changes and the macroscopic alterations in detail. Another limitation is the lack of sham operated animals, which was not included as previous studies have shown that the contralateral kidney and the sham kidneys are similar with mild ishcemia^5^.

To summarize, hyperpolarized [1–^13^C]pyruvate MRI shows great promise for future investigations in patients with renal disease, identifying the balance between the anaerobic and aerobic metabolism as a potential prognostic biomarker. The metabolic phenotype seen in mild-to-moderate ischemia induced injury is similar to the pseudo-hypoxic condition seen in diabetes^33^, which supports hypoxia as an underlying factor in the development of renal diseases and secondly represents a novel metabolic target for AKI treatment.

## Supplementary information

Supplementary information.
