# Supplementary material for: Metabolic reprogramming associated with progression of renal ischemia reperfusion injury assessed with hyperpolarized [1-13C]pyruvate
Source: Sci Rep. 2020 Jun 2;10:8915. doi: 10.1038/s41598-020-65816-1 (PMC7265284; doi:10.1038/s41598-020-65816-1)
Supplement: Supplementary file 1 — Supplementary information. [file 41598_2020_65816_MOESM1_ESM.docx]

**Metabolic reprogramming associated with progression of renal ischemia reperfusion injury assessed with hyperpolarized [1-^13^C]pyruvate**

Per Mose Nielsen^1^, Haiyun Qi^1^, Lotte Bonde Bertelsen^1^, Christoffer Laustsen^1^

^1^MR Research Centre, Department of Clinical Medicine, Aarhus University, Aarhus, Denmark

**Keywords:** MRI, hyperpolarization, acute kidney injury, Ischemia

**Running title: Hyperpolarized [1-^13^C]pyruvate can predict IRI outcome**

**Corresponding author:**

Christoffer Laustsen

Palle Juul-Jensens Boulevard 99

8200 Aarhus N

Denmark

M +45 24439141

Email: cl@clin.au.dk

**Supplemental Figure 1.** Representative hyperpolarized [1-^13^C]pyruvate and [1-^13^C]lactate images at 2 min and 60 min following reperfusion.

**Supplemental Figure 2.** Representative hyperpolarized ^13^C-Bicarbonate and [1-^13^C]lactate images at day 1 and day 7 following reperfusion.
